# Optimizing the breeding strategy in Vanaraja female parent line chicken by monitoring the genetic variability for the major performance traits

**DOI:** 10.5713/ab.25.0304

**Published:** 2025-09-01

**Authors:** Matam Niranjan, Rudra Nath Chatterjee, Aneet Kour, Lawrence Leslie Leo Prince, Santosh Haunshi, Ullengala Rajkumar

**Affiliations:** 1 Poultry Genetics and Breeding Division, ICAR-Directorate of Poultry Research, Rajendranagar, India

**Keywords:** Breeding Program, Chicken, Economic Traits, Genetic Parameters, Genetic Trend

## Abstract

**Objective:**

*Vanaraja* is a highly successful backyard chicken variety developed by crossing males of Vanaraja male line (PD-1) with females of Vanaraja female line (PD-2). *Vanaraja* was improved over the generations through selection in parent lines. This study was designed to estimate the genetic parameters and trend for major traits in PD-2 line in order to determine the future breeding strategy in the population.

**Methods:**

Seven generations data of PD-2 population was utilized to precisely examine the existing additive genetic variability for the major growth and production traits. Egg mass at 52 weeks of age (EM52) is the primary selection trait. The best-fitted model for each trait was utilized out of the six models to obtain the genetic parameters. Subsequently, estimated breeding values were used to plot genetic trend for the major production traits.

**Results:**

Models with maternal genetic and permanent environmental effects were identified as the best-fitted for most of the body weight traits. Model 1 with direct additive effect only as random effect was adjudged as the best for the primary selection trait EM52. EM52, was low heritable with the estimates as 0.13±0.03. The average estimated breeding value of EM52 increased linearly and significantly as a direct response to selection in PD-2 line and was 0.47 kg after seven generations of selection.

**Conclusion:**

Based on the findings, PD-2 population still has sufficient genetic variability for the selection trait and other performance traits. The present breeding program can continue in order to produce genetic improvement in the *Vanaraja* chicken.

## INTRODUCTION

Wide spread expansion and adoption of backyard poultry using improved germplasm bodes well for the overall socio-economic empowerment in the form of improved nutritional security, rising incomes, nurturing gender equality, health and well-being of the population [1,2]. It becomes all the more important in case of developing and under-developed, resource poor countries. In India, backyard poultry with improved chicken varieties like *Vanaraja* is being promoted by the government for sustainable livelihoods of rural and tribal communities. *Vanaraja* is a dual purpose chicken variety developed at ICAR-Directorate of Poultry Research (ICAR-DPR), Hyderabad which is perfectly suited to backyard rearing in rural and tribal areas of the country. PD-2 is the female parent of *Vanaraja* chicken which has been evolved from the synthetic multi-coloured broiler population. The continual improvement in economic traits of *Vanaraja*, the terminal cross, is being targeted through selection experiments in its parent lines.

Response to selection for any trait depends on the population size, gene frequency, mutation, allelic fixation, random drift and physiological limits [3]. Also, accurate estimation of genetic parameters at definite intervals determines the efficacy of the breeding program and dictates the prediction of the expected response in future generations, thus helping in the optimization of the breeding strategy [4]. The importance of maternal effects in development and expression of the economic traits and in realizing unbiased genetic parameters has been well documented in chicken [4,5–9]. Therefore, while estimating the additive genetic variability in a population, apportioning of the trait variance into different maternal components (in addition to the additive part) decreases the residual part, leading to precision in the estimates [10–14].

Keeping all this in consideration, the present study was conducted to determine the genetic parameters for the various growth and production traits utilizing models with different random effects in average information restricted maximum likelihood (AIREML) animal model and to see the genetic trend for the major production traits in PD-2 chicken line.

## MATERIALS AND METHODS

The study was carried out at the experimental poultry farm of ICAR-DPR, Hyderabad, Telangana, India. Hyderabad is located in Deccan plateau positioned between 17°23’ N and 78° 28’ E at a height of 500 m from mean sea level. The climate of the region is hot tropical with temperature ranging from 20°C in winter to 45°C in summer. The study was approved by Institutional Animal Ethics Committee of ICAR-DPR vide IAEC/DPR/20/7.

### Population and management

The female parent line (PD-2) of *Vanaraja* was evolved from a synthetic random-bred multi coloured broiler control populations over the generations at ICAR-DPR, Hyderabad [15,16]. Initially, the birds were under intense selection for higher egg production, however, since the last eight generations, selection criterion has been changed to higher egg mass at 52 weeks [17,18]. In this study, the data of PD-2 line spreading across past seven generations with 4–5 hatches per generation has been considered for analysis.

Population was regenerated in each generation by utilizing 50 sires and 250 dams in a pedigreed mating with one sire mated to five dams. Number of chicks ranged from 2,027 (third generation) to 3,117 (second generation). Chicks were identified by wing bands and reared on deep litter system in an open sided poultry house under standard management practices. About 473 (second generation) to 812 (sixth generation) females and 200 males were housed in individual cages. The chicks were fed *ad-libitum* with broiler starter (2,900 Kcal: ME and 22%: CP) diet based on maize-soybean meal up to 6 weeks of age followed by a broiler grower ration (2,800 Kcal: ME and 18%: CP) up to 16 weeks of age and a broiler breeder ration (2,700 Kcal: ME, 16.50%: CP and Calcium: 3.5%) up to the end of the 52 weeks of age on feed restriction schedule. The birds were vaccinated against the common diseases as per the standard schedule.

### Data and traits

The data was recorded on 17,114 chicks for juvenile growth traits (body weight and shank length up to six weeks of age) on pooled sex basis. This was due to the reason that sexing is usually done after six weeks of age in the population. The production traits were recorded for 3,602 female birds which originated from 355 sires and 1,395 dams. The juvenile data utilized for the analysis spanned all seven generations (S-1 to S-7) whereas for the adult production traits, the data was available for six generations only (S-2 to S-7). The structure and characteristics of the data with descriptive statistics of growth and production traits are presented in Table 1. Juvenile traits included body weight at 0 day (BW0), 2^nd^ (BW2), 4^th^ (BW4) and 6^th^ (BW6) week and shank length at 6^th^ week (SL6). The adult traits comprised of body weight at 20^th^ (BW20), 40^th^ (BW40) and 52^nd^ (BW52) week; age at sexual maturity (ASM), part period egg production up to 40 (EP40) and up to 52 weeks (EP52), egg weight at 24 (EW24), 28 (EW28), 32 (EW32), 36 (EW36), 40 (EW40) and 52 (EW52) weeks. Egg mass at 52 weeks (EM52) was calculated as the product of egg number and egg weight. Body weight was measured to 0.1 g accuracy using digital balance while shank length was measured to the nearest of 0.01 mm accuracy using digital Vernier Calipers. The weight of eggs was recorded using a digital balance to an accuracy of 0.01 g. The data was not available for BW0 in S-4 and S-7 and for BW2 in S-7 generation.

### Statistical analysis

Variance and covariance were estimated by AIREML fitting an animal model using WOMBAT software [19]. Data were first analyzed by least squares analysis of variance using SPSS ver. 20 software [20] to identify the fixed effects to be included in the model. Generation (6 levels) and hatch number (5 levels) were included as fixed effects in the statistical model for growth and production traits. Only significant effects (p≤0.05) were included in the models for subsequent genetic analysis. Convergence was assumed when change of value of the natural logarithm of the likelihood function in two consecutive iterations was lower than 5×10^−4^. Univariate animal models were fitted to estimate (co)variance components for all the traits. Six different models which accounted for the direct additive and maternal effects were constructed as follows:


(1) $$\text{y}=\text{X}\beta +{\text{Z}}_{\text{a}}\text{a}+ɛ$$

(2) $$\text{y}=\text{X}\beta +{\text{Z}}_{\text{a}}\text{a}+{\text{Z}}_{\text{m}}\text{m}+ɛ\;\text{with Cov }({\text{a}}_{\text{m}},{\text{m}}_{\text{o}})=0$$

(3) $$\text{y}=\text{X}\beta +{\text{Z}}_{\text{a}}\text{a}+{\text{Z}}_{\text{m}}\text{m}+ɛ\;\text{with Cov }({\text{a}}_{\text{m}},{\text{m}}_{\text{o}})=\text{A}\sigma_{\text{am}}$$

(4) $$\text{y}=\text{X}\beta +{\text{Z}}_{\text{a}}\text{a}+{\text{Z}}_{\text{pe}}\text{pe}+ɛ$$

(5) $$\text{y}=\text{X}\beta +{\text{Z}}_{\text{a}}\text{a}+{\text{Z}}_{\text{m}}\text{m}+{\text{Z}}_{\text{pe}}\text{pe}+ɛ\;\text{with Cov }({\text{a}}_{\text{m}},{\text{m}}_{\text{o}})=0$$

(6) $$\text{y}=\text{X}\beta +{\text{Z}}_{\text{a}}\text{a}+{\text{Z}}_{\text{m}}\text{m}+{\text{Z}}_{\text{pe}}\text{pe}+ɛ\;\text{with Cov }({\text{a}}_{\text{m}},{\text{m}}_{\text{o}})=\text{A}\sigma_{\text{am}}$$

Where y is the vector of records; *β*, a, m, pe and *ɛ* are vectors of fixed, direct additive genetic, maternal additive genetic, permanent environmental effects of the dam, and residual effects, respectively; with association matrices X, Z_a,_ Z_m_ and Z_pe;_ A is the numerator relationship matrix between animals; and σ_am_ is the covariance between additive direct and maternal genetic effects. Assumptions for variance (V) and covariance (Cov) matrices involving random effects were:

V(a) = Aσ^2^_a_, V(m) = Aσ^2^_m,_ V(c) = Iσ^2^_c_, V(e) = Iσ^2^_e_, and Cov(a,m) = Aσ_am,_ where I is an identity matrix and σ^2^_a,_ σ^2^_m,_ σ^2^_c_ and σ^2^_e_ are additive direct (a), additive maternal (m), maternal permanent environmental (c) and residual variances (e), respectively. Akaike’s Information Criterion (AIC) was used for selecting the best model among the tested models [21]. The model with lowest AIC value was chosen as the most appropriate model and used to study the genetic parameters.

The best models from the single trait analyses were combined with appropriate (co)variance between random effects in the model for bivariate analysis. Best model identified using likelihood ratio test, for specific trait were only used for the bivariate analysis. Genetic, phenotypic and environmental correlations between different economic traits were obtained by AIREML fitting an animal model. To formally test the significance of additive genetic correlations, the AIC for this model is compared to model in which COV_A_ = 0 is specified [22]. Significance of phenotypic correlations was tested by hypothesis test to decide whether the value of the correlation coefficient is significantly different from zero [23]. Estimated breeding values (EBVs) obtained from the best model suited for each trait was used to plot genetic trend. Genetic trend was estimated by regression of the EBV averages on generation for trait under selection (EM52) and other economically important production traits under study [23].

## RESULTS

### Least squares mean

The least squares mean (LSM) of juvenile growth traits of PD-2 line are presented in Table 2. Generation and hatch had significant (p≤0.01) effect on the body weight (BW0, BW2, BW4 and BW6) and shank length (SL6) in PD-2 line.

The LSM for growth and production traits are presented in Table 3. Generation had significant (p≤0.01) influence on adult body weights (BW20, BW40 and BW52). The adult body weights showed decreasing trend over the generations. ASM significantly (p≤0.01) varied between the generations with a decreasing trend in desired direction. Egg production (EP40 and EP52) significantly (p≤0.01) influenced by the generation effect with an increasing trend over the generations. Generation, hatch and their interaction effect was significant (p≤0.01) on egg weight at different ages (Table 4). EM52, the primary trait of selection, significantly (p≤0.01) varied between the generations and hatches. EM52 recorded an increasing trend over the generations and decreasing trend across the hatches (Table 4). The average LSM for EP52 and EM52 were 125.28±0.58 eggs and 7.37±0.03 kg, respectively in PD-2 chicken line.

### (Co)variance

The estimates of (co) variance components of juvenile traits obtained using the best model is presented in Table 5. Model 5 was the best for BW0 and model 6 for all other juvenile traits (BW2, BW4, BW6 and SL6). The best fit model for BW20 was model 4 and for BW40 and BW52 was model 6. The appropriate models for production traits were model 4 for ASM and EP52; model 1 for EP40, EW24, EW52 and EM52 respectively (Tables 6, 7).

### Heritability

The additive heritability (h^2^_a_) estimate was low to moderate in magnitude for all the body weight traits except for BW40 and BW52 which recorded higher h^2^_a_ estimates (Tables 5, 6). The maternal heritability (h^2^_m_) at birth (BW0) was moderate in magnitude which reduced gradually with age. The maternal permanent environment effect (c^2^) was also more at birth and gradually reduced as age increased with lower or negligible estimates. The h^2^_a_ estimate of ASM, EP40 and EP52 were low in magnitude (Table 6). The heritability was high in magnitude for egg weight traits with low h^2^_m_ for EW28 and very less c^2^ for EW32 and EW40, respectively. The heritability of primary trait, EM52 was 0.13±0.03 which indicates that the trait was low in magnitude (Table 7).

### Correlations

The additive genetic and environmental correlation between the juvenile growth traits is presented in Table 8. The additive genetic correlation between BW0 and other juvenile traits was significant (p≤0.01) and positive in direction, but less in magnitude (0.37±0.05, 0.23±0.06, 0.25±0.05 and 0.19±0.05 for BW0-BW2, BW0-BW4, BW0-BW6 and BW0-SL6 combinations, respectively). The additive genetic correlation coefficients between body weights (BW2, BW4, BW6) and with SL6 were highly significant (p≤0.01) with high degree of positive association (0.69±0.04, 0.84±0.03 and 0.91±0.01 respectively). The phenotypic correlation between BW2, BW4, BW6 and SL6 was positive to varying degrees (BW2-BW4 = 0.44± 0.008; BW2-BW6 = 0.55±0.007; BW2-SL6 = 0.51±0.008; BW4-BW6 = 0.53±0.007; BW4-SL6 = 0.47±0.007; BW6-SL6 = 0.84±0.003).

The additive genetic correlation between body weight (BW20, BW40 and BW52) traits was significant (p≤0.01) with high degree of positive association. The association between ASM and adult body weight traits (BW20, BW40 and BW52) and egg weights was low positive. ASM had significantly (p≤0.01) high degree of negative correlation with EP40 (−0.67±0.12) and EP52 (−0.37±0.18) (Table 9). The correlation between egg production (EP40 and EP52) and body weights traits (BW20, BW40 and BW52) was significantly negative. The egg production traits had inverse relationship with egg weight traits with significant negative correlation coefficients (Table 9). Egg production (EP40 and EP52) had significant positive relationship with egg mass trait (EM52) (0.77±0.07 and 0.84±0.04 respectively). Also, egg weight traits had positive association with egg mass at 52 weeks of age.

### Breeding value and genetic trends

**Figure 1:**
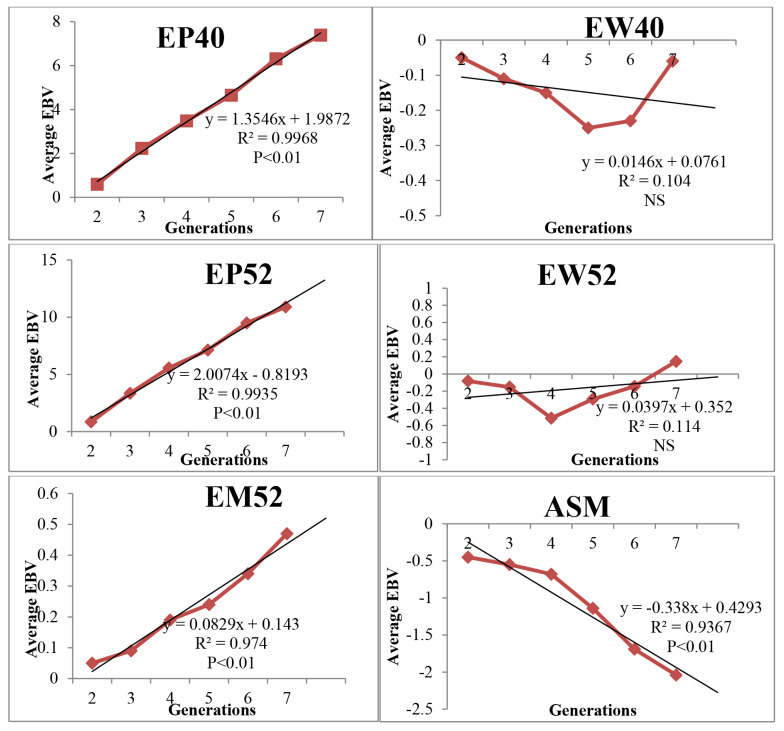
Genetic trends for various traits in PD-2 line. EBV, estimated breeding value; EP, egg production; EW, egg weight; EM, egg mass; ASM, age at sexual maturity.

**Figure 2:**
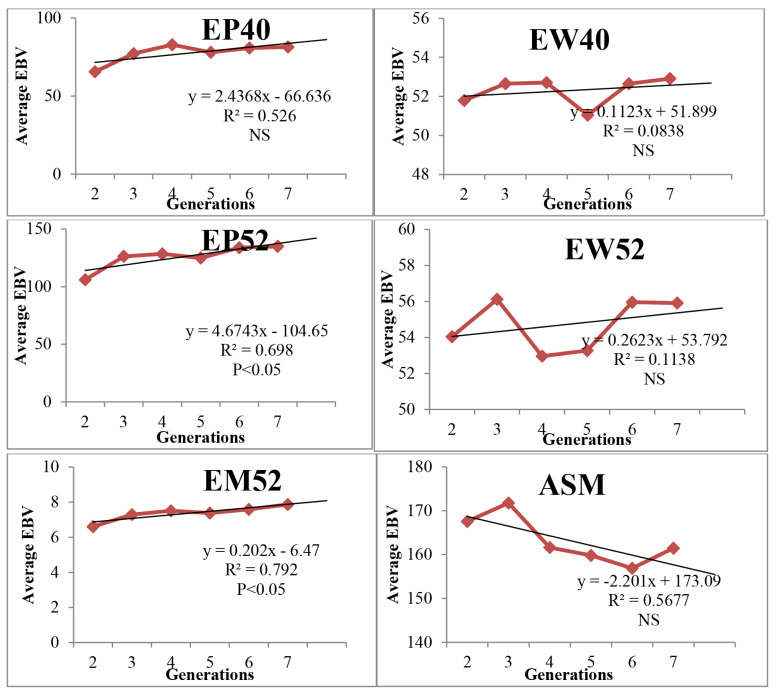
Phenotypic trends for various traits in PD-2 line. EBV, estimated breeding value; EP, egg production; EW, egg weight; EM, egg mass; ASM, age at sexual maturity.

The average EBV of EM52 increased linearly as a direct response to selection and was 0.47 kg at the end of seventh generation which was significant (p≤0.05) (Figure 1). The phenotypic trend (response) of EM52 was 7.87 kg with significant positive trend (Figure 2). The average EBV of EP52, EP40 was 10.89 and 7.39 eggs respectively after seven generations of selection. The genetic trends of egg production were significant (p≤0.05) with linear increase over the generations. The average EBV of egg weights traits (EW40 and EW52) was fluctuating with increasing trend (Figure 1). ASM reduced significantly with each generation in desired direction with average EBV of −2.04 days at the end of seven generations. The average EBV of juvenile traits (BW6 and SL6) over seven generations was 22.72 g and 0.95. The genetic trend of juvenile traits was significant (p<0.01) with linear progression.

## DISCUSSION

Body weight in early stages of life is very important for the optimum performance of the birds during the laying phase [24]. In our study, juvenile body weight linearly increased as a correlated response to selection as direct selection was not practiced for the trait. Significant generation and hatch effects on body weight and shank length were reported in PD-1 line [4,13], in PB-2 line [8]; in PD-3 line [24] and in rural parent line [25]. Faster growth rate in early part of life in female parent is a desirable feature as it aids in higher body weights in terminal crosses intended for backyard poultry farming. Interestingly, in this study, hatch effect was also showing increasing trend across the hatches, contrary to the earlier reports wherein body weight was reduced in later hatches [24]. This might be due to the better hatchery management conditions followed during the incubation of eggs and also nursery phases of chick rearing.

The decreasing trend of adult body weights over the generations might be due to the prolonged selection for egg production [17] and egg mass [18] practiced in this line, which might have impacted the body weight as both are negatively correlated traits. The variations in body weights might be attributed to the management, environment and feed restriction schedule followed during grower phase to maintain the standard body weight at laying. The BW20 was maintained at 1.8–2.0 kg, the standard body weight for synthetic female parent line at start of lay. The LSM for ASM significantly (p≤0.01) reduced over the generations in desired direction. The increased body weight during the growing phase over the generations might be one of the reasons for early maturity. Similar findings were reported by Reddy et al [26] in White Leghorn chickens where indirect selection for body weight reduced ASM. Egg production increased significantly over the generations as an indirect response to selection, as selection was practiced for egg mass in which both egg production and egg weight were considered. Similar findings of increased egg production were reported in Dahlem Red [24]. The inverse relationship between body weight and egg production in chicken was a well established fact [4,7,9,24,25,27]. Selection for higher egg mass might be responsible for maintaining egg weight over the generations and across the hatches in spite of significant increases in egg production. EM52 recorded significantly increasing trend over the generations which might be due to the higher egg production and egg weight at 52 weeks of age (Table 4). The appropriate model for all juvenile traits was model 6 with all the components included in the model sourcing the variance of different magnitudes except for BW0, wherein model 5 was the best model (Table 5). Maternal effects, both maternal genetic and maternal permanent environmental were observed in addition to the additive effects on body weights and SL up to six weeks of age in PD-2 line. However, non-significant negligible maternal effects were recorded for BW40 and BW52. These may not have any impact on the traits as their contribution was very less (0.02 for maternal genetic and 0.06 for maternal permanent environmental components). Jasouri et al [28] observed maternal genetic effects on body weight up to 12 weeks of age in dual purpose chicken and found that maternal effects decreased as age advances. Similar findings were observed in broiler crosses [12]; PD-1 [4]; in PB-2 [8] and in commercial broilers [29]. In contrary to the earlier studies, maternal effects continued till 52 weeks of age for body weight traits, however these were not significant with negligible low proportions. Similar prolonged non significant maternal effects were observed in Dahlem Red [24]. Maternal environment effects are pre- and post-ovipositional, the latter may be pre- and post-hatch effects which influence egg quality traits and chick weight at hatch [30]. The inclusion of one of the maternal effects in the model could be enough to adjust the variation occurring due to all maternal effects [19]. However, we included m, am, c effects in the model for precise variance separation and accurate estimates of genetic parameters. The inclusion of maternal effects in the model improved the robustness and goodness of fit of the model and also h^2^ estimates [31]. The importance of maternal effects for early body weight was demonstrated, though the magnitude was less than that of ‘c’ effects [5,7] in different chicken populations. The factors like hatch conditions, egg size and uterus have considerable influence on early body weight leading to high ‘c’ effects, which reduces or becomes negligible at later stages of life [4].

Model 4 was the appropriate model for ASM, EP52 and BW20 with additive and maternal permanent environmental effects, while model 1 with direct additive genetic effect only was best for EP40, EW24, EW52 and EM52 and model 3 was best for EW28, EW32 and EW40. Previously, maternal and direct additive effects were observed for the egg production and egg weight [24,28,32] similar to the present study. However, model 1 was suitable for egg production (EP40) and egg mass (EM52) traits in line with results of earlier researchers [5,33,34] in different chicken populations. The additive genetic effect increased and maternal effects decreased with age for production traits [35,36], which was true in the present study also for most of the traits.

The h^2^ estimated using animal model was more precise due to partitioning of the variance and covariance in to all possible sources. The h^2^ estimates of low magnitude with high precision and accuracy using animal model were documented in literature [4,5,7,24,25,28]. The variation in h^2^ estimates over the time was due to activation of various genes responsible for egg production [36] and persistency [37]. The lower estimates in the present study might be due to the prolonged selection for higher egg mass which reduced the variability in the population and also due to the inclusion of additional sources of variance in the model. Higher h^2^ estimates for various economic traits were observed in Mazandaran chicken [36] which may be due to lack of partitioning of the trait variance into maternal sources. Higher h^2^ estimates than the results of present study were reported for egg production in Horro chicken from Ethiopia [38] and in commercial layer line [39]. Maternal effects account for only 2–8% of variance in chicken, but ignoring these effects may lead to serious biases and overestimation of genetic parameters [33,34,40], leading to inadequate inferences hampering the breeding goals [6,32]. Similar h^2^ estimates for growth and egg production traits were reported in PD-1 [4]; in PB-2 [8]; in Dahlem Red chicken breed [24]; in Rural male parent line [25] from India using the REML with maternal effects in the model. The inclusion of maternal effects in the model led to accurate h^2^ estimates in PD-2 line which will help in planning the breeding program with reliable predictions.

Genetic correlations have important role in animal/poultry breeding as selection for one trait improves the performance in other traits as a positive response whereas negative association can reduce the trait performance [7]. The direction, magnitude and precision of correlation enables the breeder to fix the favourable traits in selection for improvement of the traits simultaneously [4]. The genetic correlation between body weight and shank length was significant and high in magnitude in PD-2 line, similar to the reports in PD-1 line [4]; in PB-2 [8]; in Mazandaran chicken [36]; in Thai native chicken [41]. The positive association between the traits aids the breeder to select the parents at a younger age reducing the input costs and economizing the returns to the farmer. The correlation between ASM and egg production (EP40 and EP52) was negative and significant (p≤0.01) in PD-2 line. Significant negative relationship between ASM and egg production was a well established fact in chickens [4,8,17,24]. Also, the birds maturing at a younger age laid smaller eggs and vice versa, as ASM and egg weights were positively correlated. The association between ASM and BW20 was weak with 0.08 as genetic correlation coefficient (r_g_). The weak positive relation/strong inverse relationship between ASM and BW20 has been documented in literature [4,11,17,24]. The inverse relationship between body weight and egg production was observed similar to the findings of many authors in chicken [7,17,24, 27]. Egg mass (EM52) had a significant positive association with egg production, positive association with egg weights, while having an antagonistic relationship with ASM. The positive association of egg mass with egg production and egg weights was natural as egg mass is the product of egg number and egg weight. Higher number of eggs coupled with better egg weight will naturally result in higher egg mass. The selection for egg mass maintains both egg number and egg weight at optimum level which is commonly practiced in breeder lines [24]. The egg mass and ASM had a low magnitude positive relationship as late matured birds recorded higher egg mass as these birds produced heavier eggs with optimum production resulting in higher egg mass.

The EBV estimation for primary trait of selection has been the important goal of the breeder for choosing the parents for next generation. The trend of EBV for EM52 over six generations was significant with positive linear trend indicating the positive selection response in the population with significant improvement. Similar positive trend for egg mass was reported in Dahlem Red chicken in our previous study [24]. The positive significant linear genetic trend was observed for egg production traits and non-significant positive trend for egg weight traits as correlated response to selection in PD-2 line. The genetic trend of BW6 and SL6 was in linear positive direction as correlated responses to selection though selection was not practiced for growth traits in the line. Similar observations of indirect response in correlated traits were reported in chicken by several workers [4,8,17,18,24]. Egg weights showed linear increasing trend with fluctuations over the generations as selection for egg mass maintained the egg weight in the population. The EBV and genetic trend observed in all the production traits was due to the correlated response to selection for egg mass as direct selection was not practiced for those traits.

## CONCLUSION

Based on the study findings, it can be concluded that the additive genetic variability for EM52 was optimal as of now, and it can be further exploited for making genetic improvements in the PD-2 chicken population.

## Figures and Tables

**Table 1:** Data structure of PD-2 chicken line

Trait	No. of birds with records	No. of sires	No. of sires with progeny	No. of dams	No. of dams with progeny	Mean±SE	CV (%)
BW0 (g)	12,846	296	254	1,078	969	37.89±0.04	10.93
BW2 (g)	14,681	304	304	1,170	1,170	121.88±0.28	27.46
BW4 (g)	17,114	355	355	1,395	1,395	285.35±0.64	29.22
BW6 (g)	16,911	355	354	1,392	1,390	553.51±1.13	26.64
SL6 (mm)	16,654	355	354	1,413	1,411	69.44±0.05	10.12
BW20 (g)	3,602	302	302	986	979	1,950±3.78	11.63
BW40 (g)	3,398	302	302	961	954	2,949.84±4.49	10.49
BW52 (g)	3,164	301	301	951	943	2,736.98±5.52	11.35
ASM (d)	3,545	302	302	983	975	162.77±0.24	8.90
EP40 (Nos.)	3,499	302	302	980	973	78.21±0.39	29.95
EP52 (Nos.)	3,425	302	302	974	967	126.66±0.59	27.22
EW24 (g)	2,205	293	284	852	796	42.32±0.08	8.48
EW28 (g)	2,979	301	301	944	923	47.07±0.06	7.14
EW32 (g)	3,060	299	299	945	933	49.05±0.06	6.48
EW36 (g)	2,976	297	297	932	923	50.76±0.06	6.55
EW40 (g)	2,970	299	298	932	918	52.39±0.06	6.25
EW52 (g)	2,679	300	300	906	893	54.99±0.08	7.29
EM52 (kg)	2,581	295	295	893	879	7.47±0.03	20.13

SE, standard error; CV, coefficient of variation; BW, body weight; SL, shank length; ASM, age at sexual maturity; EP, egg production; EW, egg weight; EM, egg mass.

**Table 2:** Least squares means (LSMs) of juvenile traits (based on pooled sex) in PD-2 line

Particulars	Body weight (g)				Shank length (mm)

	0 d	2 wk	4 wk	6 wk	6 wk
Overall LSM	37.90±0.03 (12,865)	120.16±0.19 (14,694)	272.33±2.07 (17,125)	538.46±0.95 (16,921)	68.73±0.05 (16,664)
Gen	**	**	**	**	**
1	39.50±0.07^c^ (2,457)	121.57±0.48^d^ (2,373)	287.65±1.26^c^ (2,330)	538.89±2.48^c^ (2,243)	69.88±0.13^b^ (2,234)
2	40.16±0.06^c^ (3,117)	117.91±0.48^c^ (2,379)	239.16±1.16^a^ (2,837)	469.22±2.44^a^ (2,539)	66.11±0.13^a^ (2,516)
3	37.87±0.08^b^ (2,027)	112.51±0.53^b^ (1,975)	244.63±1.39^ab^ (1,910)	484.26±2.75^b^ (1,826)	66.23±0.14^a^ (1,782)
4	-	102.14±0.46^a^ (2,733)	252.17±1.27^b^ (2,400)	470.94±2.44^a^ (2,480)	66.84±0.13^a^ (2,341)
5	34.33±0.07^a^ (2,340)	120.28±0.49^d^ (2,334)	239.32±15.13^a^ (1,744)	546.62±2.89^c^ (1,907)	67.23±0.15^a^ (1,857)
6	37.08±0.07^b^ (2,920)	146.55±0.44^e^ (2,896)	332.23±1.14^e^ (2,884)	659.83±2.20^e^ (2,899)	71.55±0.11^c^ (2,915)
7	-	-	310.03±1.20^d^ (3,016)	601.12±2.33^d^ (3,024)	72.88±0.12^c^ (3,016)
Hatch	**	**	**	**	**
1	37.54±0.06^a^ (3,343)	126.32±0.37^d^ (4,128)	310.24±0.88^e^ (4,810)	569.39±1.72^c^ (4,704)	70.57±0.09^c^ (4,681)
2	37.86±0.07^a^ (2,898)	119.22±0.45^b^ (2,675)	293.46±0.97^d^ (3,950)	545.71±1.92^b^ (3,828)	68.83±0.09^b^ (3,801)
3	38.12±0.07^b^ (2,949)	111.54±0.40^a^ (3,451)	236.14±0.99^a^ (3,799)	511.91±2.19^a^ (3,255)	67.47±0.11^a^ (3,139)
4	37.69±0.07^a^ (2,930)	120.58±0.39^c^ (3,714)	247.82±8.67^b^ (3,581)	514.44±1.90^a^ (4,178)	67.39±0.09^a^ (4,088)
5	39.89±0.13^c^ (741)	137.05±0.87^e^ (722)	278.19±2.21^c^ (981)	581.82±4.32^d^ (953)	71.02±0.22^c^ (952)
Gen×Hatch	**	**	**	**	**

Values in the parentheses are number of observations.

a–e Means with same superscripts do not differ significantly within the column separately for generation and hatch.

** p<0.01.

Gen, generation.

**Table 3:** Least squares means (LSMs) of growth and production traits in PD-2 line

Particulars	Body weight (g)			ASM (d)	EP (Nos.)	
	
	20 wk	40 wk	52 wk		40 wk	52 wk
Overall LSM	1,943.69±3.12 (3,608)	2,496.46±4.32 (3,402)	2,739.79±5.01 (3,168)	163.29±0.22 (3,551)	77.28±0.39 (3,505)	125.28±0.58 (3,429)
Gen	**	**	**	**	**	**
2	1,913.82±7.52^c^ (606)	2,629.89±11.03^e^ (511)	2,928.19±13.01^e^ (454)	167.57±0.52^d^ (595)	65.54±0.98^a^ (552)	105.97±1.48^a^ (522)
3	1,806.24±8.44^a^ (473)	2,454.58±11.31^b^ (475)	2,739.11±12.79^c^ (465)	171.77±0.58^e^ (473)	77.12±1.03^b^ (476)	126.25±1.51^b^ (476)
4	1,923.42±7.59^c^ (586)	2,385.57±10.60^a^ (552)	2,527.62±12.11^a^ (521)	161.62±0.53^c^ (569)	82.83±0.95^d^ (561)	128.41±1.42^b^ (546)
5	2,015.81±7.43^e^ (604)	2,439.49±10.19^b^ (580)	2,611.22±12.79^b^ (461)	159.82±0.52^b^ (587)	77.98±0.91^bc^ (597)	124.84±1.36^b^ (587)
6	2,172.99±8.00^e^ (527)	2,549.88±10.71^d^ (530)	2,839.22±11.92^d^ (534)	156.89±0.54^a^ (536)	80.75±0.97^cd^ (535)	133.79±1.43^c^ (536)
7	1,858.64±6.87^b^ (812)	2,488.09±9.48^c^ (754)	2,744.97±10.69^c^ (733)	161.45±0.48^c^ (791)	81.39±0.86^d^ (784)	134.88±1.28^c^ (762)
Hatch	**	^NS^	^NS^	**	**	**
1	1,971±5.98^cd^ (986)	2,494.11±8.27 (924)	2,735.72±9.74 (853)	163.12±0.41^b^ (968)	80.25±0.74^c^ (955)	128.41±1.09^c^ (939)
2	1,997.11±6.29^d^ (865)	2,503.12±8.74 (804)	2,730.22±10.04 (758)	163.70±0.43^b^ (853)	78.14±0.78^b^ (847)	127.15±1.15^c^ (827)
3	1,951.01±6.83^c^ (726)	2,510.28±9.68 (672)	2,763.69±11.12 (628)	161.04±0.47^a^ (714)	78.52±0.87^b^ (689)	125.53±1.31^b^ (669)
4	1,862.05±6.49^a^ (830)	2,474.82±8.80 (812)	2,724.45±10.23 (750)	165.83±0.45^c^ (822)	73.56±0.79^a^ (825)	122.07±1.18^ab^ (812)
5	1,922.11±12.85^b^ (201)	2,507.02±17.74 (190)	2,754.99±20.44 (179)	161.66±0.89^a^ (194)	73.26±1.62^a^ (189)	119.23±2.43^a^ (182)
Gen×Hatch	**	**	**	**	**	**

Values in the parentheses are number of observations.

a–e Means with same superscripts do not differ significantly within the column separately for generation and hatch.

** p<0.01.

ASM, age at sexual maturity; EP, egg production; Gen, generation; NS, non-significant.

**Table 4:** Least squares means (LSMs) of egg weight (EW) and egg mass (EM) traits in PD-2 line

Particulars	EW (g)						EM (kg)

	24 wk	28 wk	32 wk	36 wk	40 wk	52 wk	52 wk
Overall LSM	41.89±0.09 (2,209)	46.91±0.14 (2,984)	49.09±0.06 (3,064)	50.64±0.06 (2,980)	52.29±0.06 (2,974)	54.73±0.08 (2,683)	7.37±0.03 (2,585)
Gen	**	**	**	**	**	**	**
2	41.79±0.23^b^ (266)	46.60±0.16^a^ (456)	49.19±0.17^b^ (391)	50.30±0.17^a^ (384)	51.79±0.16^a^ (434)	54.04±0.21^b^ (338)	6.61±0.83^a^ (308)
3	40.32±0.38^a^ (134)	46.71±0.16^ab^ (409)	48.86±0.14^ab^ (467)	50.64±0.15^a^ (455)	52.65±0.15^b^ (433)	56.12±0.19^c^ (406)	7.29±0.70^b^ (403)
4	41.28±0.20^b^ (309)	46.38±0.82^a^ (387)	49.07±0.14^b^ (512)	51.17±0.14^b^ (504)	52.71±0.15^b^ (461)	52.96±0.21^a^ (343)	7.51±0.78^bc^ (332)
5	43.19±0.17^c^ (394)	47.67±0.14^b^ (512)	49.38±0.14^b^ (492)	50.32±0.15^a^ (458)	51.04±0.17^a^ (418)	53.27±0.19^a^ (407)	7.38±0.70^b^ (401)
6	42.81±0.15^c^ (525)	47.53±0.14^b^ (535)	48.49±0.13^a^ (533)	50.28±0.14^a^ (535)	52.65±0.14^b^ (535)	55.96±0.16^c^ (535)	7.59±0.62^bc^ (519)
7	41.93±0.17^b^ (581)	46.72±0.14^ab^ (685)	49.48±0.13^c^ (669)	51.13±0.14^b^ (644)	52.91±0.13^b^ (693)	55.91±0.16^c^ (654)	7.87±0.60^c^ (622)
Hatch	^NS^	**	**	**	**	^NS^	**
1	42.05±0.22^b^ (647)	47.23±0.11^b^ (863)	48.71±0.11^a^ (873)	50.64±0.13^b^ (784)	51.91±0.11^a^ (816)	55.05±0.15 (722)	7.60±0.58^b^ (695)
2	41.71±0.19^a^ (513)	46.89±0.55^ab^ (659)	48.92±0.12^ab^ (710)	51.24±0.12^c^ (742)	52.32±0.12^a^ (737)	54.75±0.15 (625)	7.49±0.59^b^ (603)
3	41.90±0.16^b^ (510)	47.11±0.13^b^ (636)	49.05±0.14^b^ (591)	49.54±0.14^a^ (585)	52.21±0.15^a^ (525)	54.57±0.18 (522)	7.48±0.68^b^ (500)
4	41.77±0.19^a^ (443)	46.56±0.13^a^ (674)	49.52±0.12^bc^ (728)	50.94±0.12^b^ (727)	52.49±0.12^ab^ (728)	54.51±0.15 (667)	7.04±0.57^ab^ (647)
5	42.21±0.36^c^ (96)	46.56±0.26^a^ (152)	49.68±0.24^c^ (162)	51.29±0.27^c^ (142)	53.03±0.24^b^ (168)	54.81±0.31 (147)	6.88±0.12^a^ (140)
Gen×Hatch	**	**	**	**	**	**	**

Values in the parentheses are number of observations.

a–c Means with same superscripts do not differ significantly within the column separately for generation and hatch.

** p<0.01.

Gen, generation; NS, non-significant.

**Table 5:** (Co)variance estimates of juvenile traits obtained using best fit models in PD-2 line

Trait	BW0	BW2	BW4	BW6	SL6
Best model	Model 5	Model 6	Model 6	Model 6	Model 6
σ^2^_a_	1.07	129.16	610.64	2,841.12	5.99
σ^2^_m_	1.76	8.49	103.94	207.84	0.41
σ_am_	0	−28.48	−219.64	−768.43	−1.53
σ^2^_c_	2.32	45.05	233.78	867.67	1.98
σ^2^_e_	7.82	728.64	4,331.40	13,548.50	34.41
σ^2^_P_	12.97	882.86	5,060.11	16,696.70	41.26
h^2^_a_±SE	0.08±0.03	0.15±0.03	0.12±0.02	0.17±0.03	0.15±0.03
h^2^_m_±SE	0.14±0.03	0.01±0.01	0.02±0.01	0.01±0.01	0.01±0.01
c^2^±SE	0.18±0.03	0.05±0.01	0.05±0.01	0.05±0.01	0.05±0.01
AIC	−20,977.23	−56,762.38	−81,216.33	−90,207.27	−38,949.06

BW, body weight; SL, shank length; σ^2^_a_, additive genetic variance; σ^2^_m_, maternal genetic variance; σ_am_, genetic covariance between additive and maternal genetic variance; σ^2^_c_, maternal permanent environmental variance; σ^2^_e_, residual variance; σ^2^_P_, phenotypic variance; h^2^_a_±SE, direct additive heritability±standard error; h^2^_m_±SE, maternal genetic heritability±standard error; c^2^±SE, σ^2^_mpe_/σ^2^_P_; AIC, Akaike information criterion.

**Table 6:** (Co)variance estimates of growth and production traits obtained using best fit models in PD-2 line

Trait	BW20	BW40	BW52	ASM	EP40	EP52
Best model	Model 4	Model 6	Model 6	Model 4	Model 1	Model 4
σ^2^_a_	7,250.64	21,990.2	29,573.5	24.07	62.91	113.63
σ^2^_m_		1,223.88	1,111.02			
σ_am_		−5,121.64	−5,747.51			
σ^2^_c_	2,624.86	3,858.94	4,550.69	9.17		27.02
σ^2^_e_	28,294	41,095.8	50,542.1	150.22	450.17	955.88
σ^2^_P_	38,169.5	68,168.82	85,777.31	183.46	513.08	1,096.53
h^2^_a_±SE	0.19	0.35±0.07	0.37±0.07	0.13±0.03	0.12±0.03	0.10±0.03
h^2^_m_±SE		0.02±0.03	0.01±0.03			
c^2^±SE	0.07	0.06±0.02	0.06±0.02	0.05±0.02		0.03±0.02
AIC	−20,668.47	−20,327.26	−19,292.81	−10,952.63	−12,627.57	−13,659.24

BW, body weight; ASM, age at sexual maturity; EP, egg production; σ^2^_a_, additive genetic variance; σ^2^_m_, maternal genetic variance; σ_am_, genetic covariance between additive and maternal genetic variance; σ^2^_c_, maternal permanent environmental variance; σ^2^_e_, residual variance; σ^2^_P_, phenotypic variance; h^2^_a_±SE, direct additive heritability±standard error; h^2^_m_±SE, maternal genetic heritability±standard error; c^2^±SE, σ^2^_mpe_/σ^2^_P_; AIC, Akaike information criterion.

**Table 7:** (Co)variance estimates of egg weight (EW) and egg mass (EM) traits obtained using best fit models in PD-2 line

Trait	EW24	EW28	EW32	EW40	EW52	EM52
Best model	Model 1	Model 3	Model 3	Model 3	Model 1	Model 1
σ ^2^_a_	3.51	4.05	2.94	3.48	5.06	0.27
σ ^2^_m_		0.53				
σ_am_		−0.67				
σ ^2^_c_			0.63	0.41		
σ ^2^_e_	8.99	7.37	7.27	6.65	9.49	1.76
σ ^2^_P_	12.5	11.95	10.84	10.54	14.55	2.04
h^2^_a_±SE	0.281±0.04	0.36±0.07	0.27±0.04	0.33±0.04	0.35±0.04	0.13±0.03
h^2^_m_±SE		0.05±0.03				
c^2^±SE			0.06±0.02	0.04±0.02		
AIC	−3,832.27	−4,998.64	−4,927.83	−4,853.48	−4,818.04	−2,202.67

σ^2^_a_, additive genetic variance; σ^2^_m_, maternal genetic variance; σ_am_, genetic covariance between additive and maternal genetic variance; σ^2^_c_, maternal permanent environmental variance; σ^2^_e_, residual variance; σ^2^_P_, phenotypic variance; h^2^_a_±SE, direct additive heritability±standard error; h^2^_m_±SE, maternal genetic heritability±standard error; c^2^±SE, σ^2^_mpe_/σ^2^_P_; AIC, Akaike information criterion.

**Table 8:** Additive genetic and environmental correlation between different juvenile traits in PD-2 line

	BW0	BW2	BW4	BW6	SL6
BW0		0.37±0.05**	0.23±0.06**	0.25±0.05**	0.19±0.05**
BW2	−0.08±0.02		0.81±0.03**	0.76±0.03**	0.69±0.04**
BW4	−0.00±0.02	0.34±0.01		0.91±0.02**	0.84±0.03**
BW6	−0.04±0.02	0.48±0.01	0.43±0.01		0.91±0.01**
SL6	−0.03±0.02	0.46±0.01	0.38±0.01	0.82±0.004	

Values above the diagonal indicate additive genetic correlation between the traits and values below the diagonal indicate environmental correlation between the traits.

** p<0.01.

BW, body weight; SL, shank length.

**Table 9:** Additive genetic and environmental correlation between different production traits in PD-2 line

	BW20	BW40	BW52	ASM	EP40	EP52	EW24	EW28	EW32	EW36	EW40	EW52	EM52
BW20		0.74± 0.08**	0.74± 0.08**	0.08± 0.16	−0.17± 0.15**	−0.19± 0.18	0.31± 0.13*	0.09± 0.10	0.31± 0.13	0.12± 0.15	0.19± 0.09*	0.11± 0.12	−0.13± 0.15
BW40	0.30± 0.03		0.97± 0.03**	0.42± 0.15**	−0.27± 0.14**	−0.24± 0.17**	0.26± 0.11**	0.14± 0.09**	0.24± 0.13**	0.22± 0.09**	0.27± 0.11**	0.28± 0.10**	−0.04± 0.14
BW52	0.31± 0.03	0.59± 0.02		0.24± 0.11**	−0.14± 0.14**	−0.01± 0.17**	0.26± 0.11**	0.22± 0.12*	0.25± 0.12	0.17± 0.09**	0.15± 0.09**	0.23± 0.09**	−0.05± 0.13
ASM	−0.43± 0.03	−0.03± 0.03	−0.11± 0.03		−0.67± 0.12**	−0.37± 0.18	0.02± 0.16	0.28± 0.13	0.24± 0.12	0.28± 0.11**	0.10± 0.14**	0.25± 0.13**	−0.12± 0.15
EP40	0.25± 0.03	−0.12± 0.03	−0.05± 0.03	−0.37± 0.02		0.93± 0.03**	−0.17± 0.14*	−0.04± 0.14	−0.18± 0.12*	−0.44± 0.12**	−0.34± 0.12	−0.28± 0.12*	0.77± 0.07**
EP52	0.16± 0.03	−0.07± 0.03	−0.05± 0.03	−0.22± 0.02	0.85± 0.01		−0.20± 0.15**	0.01± 0.16	−0.07± 0.18**	−0.44± 0.15**	−0.38± 0.15**	−0.27± 0.12**	0.84± 0.04**
EW24	0.11± 0.04	−0.05± 0.04	0.00± 0.05	−0.19± 0.04	0.09± 0.04	0.06± 0.04		0.87± 0.07**	0.77± 0.08**	0.75± 0.08**	0.69± 0.08**	0.59± 0.09**	0.17± 0.15
EW28	0.08± 0.03	0.05± 0.04	0.03± 0.04	0.04± 0.03	0.00± 0.03	0.00± 0.03	0.14± 0.04		0.97± 0.04**	0.90± 0.06**	0.93± 0.05**	0.77± 0.08**	0.29± 0.15
EW32	−0.03± 0.03	0.06± 0.03	0.07± 0.04	0.09± 0.03	−0.03± 0.03	−0.02± 0.03	0.13± 0.04	0.34± 0.03		0.97± 0.03**	0.95± 0.04**	0.84± 0.06**	0.32± 0.14
EW36	0.08± 0.03	0.05± 0.03	0.03± 0.04	0.34± 0.16	0.02± 0.03	0.04± 0.03	0.15± 0.04	0.22± 0.03	0.33± 0.03		0.95± 0.04**	0.84± 0.06**	0.03± 0.14
EW40	0.05± 0.04	0.11± 0.04	0.12± 0.04	0.07± 0.03	−0.02± 0.03	0.01± 0.03	0.13± 0.05	0.22± 0.04	0.34± 0.03	0.36± 0.03		0.93± 0.04**	0.05± 0.13
EW52	0.05± 0.04	0.09± 0.04	0.13± 0.04	0.00± 0.03	−0.04± 0.03	−0.05± 0.03	0.12± 0.05	0.19± 0.04	0.28± 0.03	0.32± 0.03	0.37± 0.03		0.20± 0.13
EM52	0.21± 0.03	−0.01± 0.03	0.02± 0.03	−0.35± 0.03	0.81± 0.01	0.95± 0.003	0.07± 0.04	0.06± 0.03	0.06± 0.03	0.09± 0.03	0.13± 0.04	0.23± 0.03	

Values above the diagonal indicate additive genetic correlation between the traits and values below the diagonal indicate environmental correlation between the traits.

* p<0.05,

** p<0.01.

BW, body weight; ASM, age at sexual maturity; EP, egg production; EW, egg weight; EM, egg mass.

## Data Availability

Upon reasonable request, the datasets of this study can be available from the corresponding author.
